# An Ecchymosis with Fulminant Evolution

**DOI:** 10.3390/dermatopathology8040057

**Published:** 2021-12-11

**Authors:** Andrea Michelerio, Stefania Barruscotti, Nathalie Rizzo, Carlo Tomasini

**Affiliations:** 1 Dermatology Clinic, Fondazione IRCCS Policlinico San Matteo, 27100 Pavia, Italy; andrea.michelerio01@universitadipavia.it (A.M.); s.barruscotti@smatteo.pv.it (S.B.); 2 Department of Clinical-Surgical, Diagnostic and Pediatric Sciences, University of Pavia, 27100 Pavia, Italy; 3 Department of Pathology, IRCCS San Raffaele Scientific Institute, 20132 Milan, Italy; rizzo.nathalie@hsr.it

**Keywords:** angiosarcoma, vascular tissue neoplasm, hemangiosarcoma, hemangiosarcoma/mortality

## Abstract

We present the case of an 83-year-old woman who presented with an advanced cutaneous angiosarcoma of the head. The tumor had first appeared as a small ecchymosis on her forehead 3 months before admission. There was an extremely rapid and dramatic evolution, as evidenced by photographic documentation by her relatives. Unfortunately, the delay in access to the healthcare system due to the COVID-19 pandemic lockdown and the fulminant growth were the main determinants for our patient outcome.

## 1. Introduction

Angiosarcomas are rare and malignant soft-tissue sarcomas of endothelial cell origin with a poor prognosis. Tumors may develop in any organ, with a predilection for the skin of the head, neck, and scalp of elderly men. Chronic lymphoedema and therapeutic radiation are the most common risk factors, hence secondary breast angiosarcomas are an important subgroup [1].

## 2. Case Report

In December 2020, an 83-year-old woman presented to the Emergency Department of our hospital with a large ulcerated and necrotic bulging lesion on her forehead. Ill-defined, dusky erythematous plaques extended on the parietal and frontal areas of the scalp and the face. Violaceous-darkish nodules were also observed. Comorbidities included chronic obstructive pulmonary disease, hypertension, diabetes, and ischemic encephalopathy. The physical examination revealed bilateral cervical lymphadenopathy.

**Figure 1:**
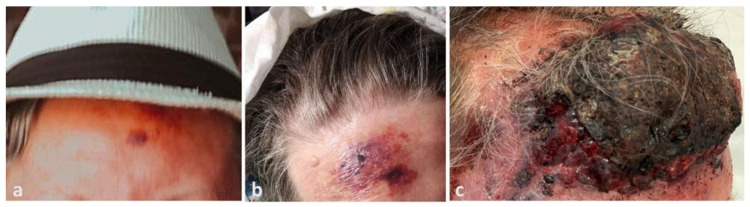
(**a**) Dramatic progression of angiosarcoma of the scalp, beginning as a small erythemato-violaceous patch on the patient’s forehead three months before admission, (**b**) extending to a large violaceous plaque on the patient’s forehead one month later, (**c**) and finally becoming a large, raised, necrotic and bleeding mass on the forehead with infiltrative plaques and nodules on the face three months later.

The patient’s relatives provided photographic documentation of the evolution. The lesion had emerged four months before admission as a 2 cm bruise-like patch on the forehead (Figure 1a), before it rapidly developed into a large purplish plaque after 1 month (Figure 1b), and then to the current presentation (Figure 1c).

**Figure 2:**
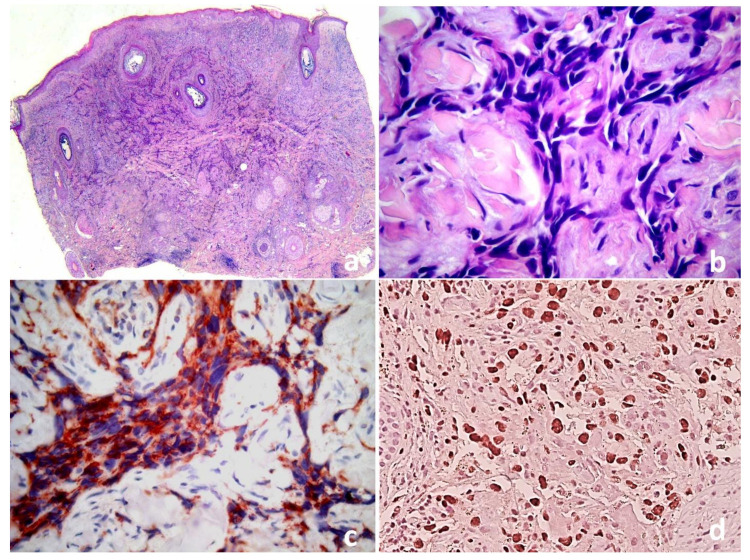
(**a**) Cutaneous angiosarcoma: proliferation of irregular vessels dissecting through the dermis. (**b**) Higher magnification demonstrates slit-like irregular vessels lined by atypical endothelial cells.The endothelial differentiation of malignant cells is demonstrated by CD31 cytoplasmic (**c**) and ERG nuclear expression (**d**) (IHC, 10×).

The second lockdown in Italy and the fear of the SARS-CoV-2 contagion had led the relatives to postpone the medical evaluation. A biopsy from a violaceous nodule showed a full dermal proliferation of irregular anastomosing vascular channels lined by single or double layers of enlarged endothelial cells, which permeated between collagen bundles, causing “collagen dissection” (Figure 2a,b). The endothelial cells were large and pleomorphic, with vesicular nuclei and prominent nucleoli, and were immunoreactive for CD31, CD34 and ERG (Figure 2c,d), with no observed HHV8 expression or MYC overexpression.

These data confirmed the diagnosis of angiosarcoma of the scalp. All routine investigations were normal. Total body computed tomography (CT) showed cervical lymphadenopathy without brain or visceral metastases. Although radiotherapy and electrochemotherapy were considered, they were not performed due to the patient’s advanced age, comorbidities, and tumor size. The patient was referred to palliative care.

## 3. Discussion

Primary cutaneous angiosarcoma (cAS) is one of the most aggressive skin tumors with a dismal prognosis [1,2]. Its initially indolent clinical presentation explains the frequently delayed diagnosis that, together with its typical multifocal pattern and poor demarcation, often makes surgery difficult [2,3]. Three main clinical variants can be identified: idiopathic angiosarcoma of the scalp and face of the elderly, angiosarcoma associated with chronic lymphedema (Stewart–Treves syndrome), and post-irradiation angiosarcoma [1,4]. The disease typically presents with bruise-like purple macules and papules that rapidly evolve into nodules and plaques. 

Histopathologically, the tumor may show irregular anastomosing vascular channels lined by a single layer of enlarged endothelial cells between collagen bundles, figuratively referred to as the dissection of collagen. Nuclear atypia and mitotic figures are invariably present, and a high mitotic rate correlates with a poor prognosis. In less differentiated tumors, little or no evidence of luminal differentiation can simulate other neoplasias and immunohistochemistry is required. This is especially the case for ERG and CD31, which are more sensitive vascular markers than CD34 [5]. There are no reliable histologic differences among the clinical subtypes of angiosarcomas.

The differential diagnosis of cAS includes multiple entities; Kaposi sarcoma is one of the most important. In fact, the histopathological features of Kaposi sarcoma (KS) and cAS may be very similar. Both neoplasms form slit-like vascular structures and may demonstrate spindle cell morphology [6]. The characteristic promontory sign (blood vessels protruding into an abnormal vascular space), although often seen in KS, is not specific. Similarly, “dissection of collagen” can be demonstrated in Kaposi sarcoma or even in benign lymphangioendothelioma [6]. However, nuclear atypia is usually less prominent in KS. Nuclear expression of HHV-8 by immunohistochemistry is seen in KS but not in cAS [6].

The American Joint Committee on Cancer (AJCC) TNM staging system for soft tissue sarcomas is not applicable to cAS, and the histopathological grading system is not prognostic for cAS [3]. Moreover, as there is no standardized treatment algorithm for each stage, the staging of cutaneous angiosarcoma has little clinical benefit in the treatment decision. In fact, the prognosis of cAS is poor, with a high rate of local recurrence (44–100%) and a tendency to metastasize despite aggressive therapies [2,3]. Tumor size, patient age, localization on the scalp, positive surgical margins, and tumor recurrence, as well as regional or distant involvement, are predictors of poor prognosis [3,7,8]. Although no clear guidelines exist, the achievement of 3 cm or greater clear margins, as well as deep margins, is recommended [2]. 

Unfortunately, despite the consensus in the literature that angiosarcoma is a fast-growing tumor, there is rarely any objective data about the growth rate. A series of 47 cases where the lesions had appeared 1 to 3 months earlier reported that 95% were on the scalp and neck, and they were on average 5.3 cm (range: 1.1–8.9 cm) [3]. Unfortunately, the mean time of onset is not reported for each lesion, so it is not possible to extrapolate an average growth rate. The lesion documented in Figure 1a is much smaller than the reported average and might be the smallest angiosarcoma ever clinically photographed. The photo sequence documents the rapid growth, eliminating a possible recall bias. Despite the evolution, MYC was not overexpressed. This is somehow expected since data in the literature have already demonstrated that the MYC abnormalities, present in a subset of primary cAS, do not seem to be related to proliferation index, histopathological, or clinical variables; thus, the significance of this finding is unclear [9,10]. 

First line treatment is radical surgery with at least 3 cm margins and adjuvant radiotherapy since the tumor extends well beyond the limits of the apparent clinical lesion, and multifocality is frequent [2]. If surgery is contraindicated or visceral metastases have already developed, chemotherapy with paclitaxel and/or doxorubicin may be indicated but provides modest results [11,12]. Electrochemotherapy may be a valid option or serve as neoadjuvant therapy prior to surgical excision [12,13].

## 4. Conclusions

In conclusion, this case documents the clinical presentation of AS of the scalp and face at a very early stage, in addition to its rapid evolution and aggressiveness. As a clear correlation exists between the prognosis and the size of AS, a high index of suspicion is paramount when dealing with ecchymoses of recent onset, especially without a history of trauma, on the face or scalp of old people.

## Data Availability

Data are contained within the article.
